# Cancer risks among patients with type 2 diabetes: a 10-year follow-up study of a nationwide population-based cohort in Taiwan

**DOI:** 10.1186/1471-2407-14-381

**Published:** 2014-05-29

**Authors:** Cheng-Chieh Lin, Jen-Huai Chiang, Chia-Ing Li, Chiu-Shong Liu, Wen-Yuan Lin, Teng-Fu Hsieh, Tsai-Chung Li

**Affiliations:** 1 Department of Family Medicine, China Medical University Hospital, Taichung, Taiwan; 2 School of Medicine, College of Medicine, China Medical University, Taichung, Taiwan; 3 Department of Medical Research, China Medical University Hospital, Taichung, Taiwan; 4 Graduate Institute of Biostatistics, College of Management, China Medical University, 91 Hsueh-Shih Road, Taichung 40421, Taiwan; 5 Health Promotion Administration, Ministry of Health and Welfare, Taipei, Taiwan; 6 Division of Urology, Department of Surgery, Buddhist Tzu Chi General Hospital, Taichung Branch, Taichung, Taiwan; 7 School of Medicine, Buddhist Tzu Chi University, Hualien, Taiwan; 8 Graduate Institute of Clinical Medical Science, College of Medicine, China Medical University, Taichung, Taiwan; 9 Department of Healthcare Administration, College of Health Science, Asia University, Taichung, Taiwan

**Keywords:** T2DM, Cancer risks, Liver cancer, Colorectal cancer, Pancreas cancer, Breast cancer

## Abstract

**Background:**

This study aims to determine cancer risks among patients with type 2 diabetes through a follow-up study on a nationwide population-based cohort that included Taiwanese diabetic patients and general population in Taiwan as well as to estimate the population attributable fraction (PAF) of site-specific cancer risks that can be attributed to type 2 diabetes in Taiwanese population by using standardized incidence ratios (SIRs, 95% CI).

**Methods:**

**S**ubjects with type 2 diabetes consisted of 472,979 patients aged ≥20 years, whereas general population consisted of 9,411,249 individuals of the same age limit but are not diabetic. Subjects were identified from 1997 to 1998 and followed up until December 31, 2007 or until the first manifestation of any cancer.

**Results:**

Cancer sites with increased risks in men, which were consistent with the main and sensitivity analyses, included pancreas (SIR = 1.62; 95% CI = 1.53 to 1.72), liver (1.61; 1.57 to 1.64), kidney (1.32; 1.25 to 1.40), oral (1.16, 1.12 to 1.21), and colorectal (1.19, 1.15 to 1.22). Cancer sites with increased risks in women included liver (1.55; 1.51 to 1.60), pancreas (1.44; 1.34 to 1.55), kidney (1.38; 1.30 to 1.46), endometrium (1.36; 1.26 to 1.47), bladder (1.19; 1.11 to 1.27), colorectal (1.16; 1.13 to 1.20), and breast (1.14; 1.09 to 1.18). Overall, PAFs were highest for liver cancer in men (4.0%) and women (3.7%), followed by pancreas (3.4%) and kidney (1.6%) cancers in men, and then for endometrium (1.8%) and kidney (1.8%) cancers in women.

**Conclusion:**

Our data suggested that increased cancer risks are associated with type 2 diabetes.

## Background

Diabetes is one of major public health problems in the world. The prevalence of type 2 diabetes mellitus has rapidly increased in Asian populations because of Westernized lifestyle behaviors [1]. Diabetes mellitus (DM) is also one of health burdens in Taiwan, and it ranks fifth among the top 10 leading causes of deaths in 2009. According to Taiwan National Health Insurance Research Database (NHIRD), age-standardized prevalence rates of type 2 diabetes have increased from 5.7% to 8.6% for men and from 5.9% to 8.0% for women from 2000 to 2007 [2]. In addition, new type 2 diabetes cases in younger adult population have increased [3]. Prevalence of diabetes is also indicated in the Taiwanese Survey on Hypertension, Hyperglycemia, and Hyperlipidemia, where diabetes incidence is 7.5% in male and 6.8% in female from 2002 to 2007 [4].

Epidemiological findings of cohort and case–control studies have reported possible association between type 2 diabetes and several cancer types, which include colon [5,6], liver [7,8], pancreatic [9,10], breast [11] and prostate cancers [12,13]. DM and cancers have common risk factors, such as smoking, alcohol consumption, obesity, diet, physical inactivity, high calorie intake, and saturated fat intake [14]. Moreover, several possible biological mechanisms that are likely involved in the association between diabetes and cancer have been proposed [15-17].

Previous studies have reported on estimated standardized incidence ratios (SIRs) by adjusting population structure for site-specific cancers in patients with DM, including those in Sweden [6,18], China [18], USA [5], and Denmark [19]. SIRs are useful for researchers, policy-makers, and health-care planners to describe the health status of a given population for planning necessary medical care services. However, studies on estimating SIRs for all site-specific cancers in Taiwanese have never been conducted. Several studies on the association of type 2 diabetes with cancers in Taiwan have focused on one specific cancer site, such as the prostate [20], colon [21], liver [22], and breast [23]. However, none of these studies have considered all cancer types simultaneously. Thus, the present study specifically aims to estimate cancer risks among patients with type 2 diabetes through a follow-up study on a national population-based cohort that include all Taiwanese diabetic patients and general population in Taiwan as well as to estimate population attributable fractions (PAF) of site-specific cancer risks in Taiwan population that can be attributed to type 2 diabetes by using SIRs .

## Methods

### Data sources

A national health insurance program was implemented in March 1995 [24]. In 2007, 22.6 million individuals from a total population of 23.0 million in Taiwan were enrolled in this insurance program. The Bureau of National Health Insurance (BNHI) contracted with 97% of hospitals and 92% of clinics in Taiwan. The datasets of the study consisted of registry for beneficiaries, ambulatory and inpatient care claims, and Registry for Catastrophic Illness from 1996 to 2007 from NHIRD. BNHI performs quarterly expert reviews on random samples of every 50 to 100 ambulatory and inpatient claims in each hospital and clinic. False diagnosis reports entail a high penalty.

**Figure 1:**
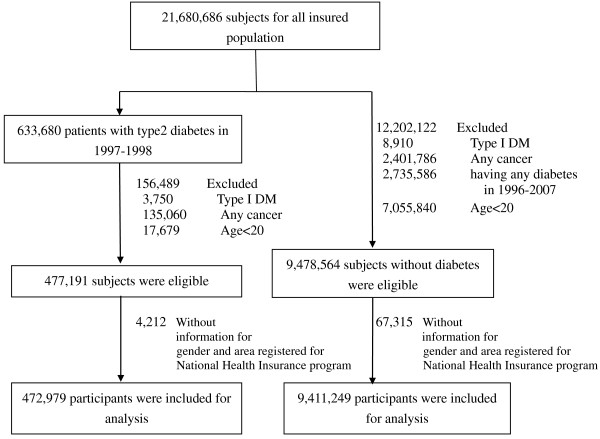
Flowchart of recruitment procedures for the current study.

Every individual in Taiwan has a unique personal identification number (PIN) code. To protect privacy, data on patient identities are scrambled cryptographically by NHIRD. All the datasets can be interlinked through each individual PIN. Ambulatory care claims contain individual’s gender and birthday, date of visit, and codes for the International Classification of Diseases, Ninth Revision, Clinical Modification (ICD-9-CM) codes, or A-codes for three primary diagnoses. Inpatient claims contain ICD-9-CM codes for principal diagnosis up to four secondary diagnoses. Registry for Catastrophic Illness database contains data from insurers who suffer from major diseases and are granted exemption from co-payment. All cancer cases registered in the catastrophic illness database should be confirmed by pathological reports. Our study using these data was exempted from institutional review board approval of Public Health, Social and Behavioral Science Committee Research Ethics Committee, China Medical University and Hospital.We conducted a population-based cohort study of two groups. Patients with type 2 diabetes (aged ≥ 20 years) were identified in 1997 to 1998 and followed up until December 31, 2007 or until the first manifestation of any cancer type. Population with type 2 diabetes should have at least three ambulatory claims or at least one inpatient claim with diagnosis of ICD-9-CM code 250 or A-code A181 from 1997 to 1998. To exclude those individuals with type 1 diabetes, we have done two steps. First, we identify all individuals with type 1 diabetes from Registry for Catastrophic Illness database. Second, we excluded those individuals with type 1 diabetes identified in the first step from our study cohort with diabetes. We initially excluded subjects with type 1 diabetes (N = 3,750), any cancer type (N = 135,060), and those aged <20 years (N = 17,679) at baseline from 633,680 patients with type 2 diabetes aged ≥ 20 years. We further excluded those with incomplete information for gender and area registered for NIH program (N = 4,212). The other group was general population, which comprised all insured individuals of the same age without any diabetes. The baseline date or index date for type 2 diabetes group was date of the first outpatient visit or inpatient admission. For general population, the index date was randomly assigned between January 1, 1997 and December 31, 1998 according to index date distribution of type 2 diabetes group. A total of 21,680,686 subjects were obtained from insured population in the data (Figure 1), and we excluded subjects with type 1 diabetes (N = 8,910), any cancer at baseline (N = 2,401,786), any diabetes (N = 2,735,586) from 1996 to 2007, and individuals aged < 20 years (N = 7,055,840). Those with incomplete information for gender and area registered for NIH program (N = 67,315) from 1997 to 1998 were also excluded. Thus, 472,979 patients with type 2 diabetes and 9,411,249 individuals without any diabetes from 1997 to 1998 were included in final analysis.

### Measurements

Sociodemographic factors include age, gender, insurance premium, and urbanization degree of area registered for NIH program. Age was divided into 17 groups with five-year intervals from 20 to >90 years. Gender was categorized into male and female. Insurance premium was categorized according to median of the amounts of insurance premiums, in which median value for these two groups was both 19,200 NT dollars from 1997 to 1998. We used an urbanization indicator developed by Liu et al. [25], who categorized 365 Taiwan towns into seven degrees of urbanization: high- and medium-density urban areas, newly developed area, general area, aging-society area, rural area, and non-developed area.

Cancer cases were identified from ambulatory and inpatient care claims of NHIRD from 1999 to 2007. Incidence rates of lung cancer (ICD-9 code 162; A-code A101), liver cancer (ICD-9 codes 155; A-code A095), colorectal cancer (ICD-9 codes 153, 154; A-code A093, A094), breast cancer (ICD-9 code 174; A-code A113), gastric cancer (ICD-9 code 151; A-code A091), oral cancer (ICD-9 codes 140 to 141, 143 to 146, 148 to 149; A-code A08), prostate cancer (ICD-9 code 185; A-code A124), esophageal cancer (ICD-9 code 150; A-code A090), pancreatic cancer (ICD-9 code 157; A-code A096), cervical cancer (ICD-9 codes 179, 180; A-code A120), nasopharyngeal cancer (ICD-9 code 147; A-code A08-01), small intestine, including duodenum cancer (ICD-9 code 152; A-code A092), gallbladder cancer (ICD-9 code 156; A-code A099-02), retroperitoneum and peritoneum cancers (ICD-9 code 158; A-code A099), laryngeal cancer (ICD-9 code 161; A-code A100), respiratory and intrathoratic organ cancers (ICD-9 codes 160, 163 to 165; A-code A109), bone cancer (ICD-9 code 170; A-code A110), connective and other soft tissue cancers (ICD-9 code 171; A-code A114), skin cancer (ICD-9 code 172; A-code A111), placenta cancer (ICD-9 code 181; A-code A121), endometrial cancer (ICD-9 code 182; A-code A122-01), ovarian cancer (ICD-9 code 183; A-code A123), testicular cancer (ICD-9 code 186; A-code A125), penile cancer (ICD-9 code 187; A-code A129-02), bladder cancer (ICD-9 code 188; A-code A126), kidney cancer (ICD-9 code 189; A-code A129-04), brain cancer (ICD-9 code 191; A-code A130), Hodgkin’s disease (ICD-9 code 201; A-code A140), leukemia (ICD-9 codes 204 to 208; A-code A141), and carcinoma in situ (ICD-9 codes 230 to 234; A-code A16) were estimated for type 2 diabetes group and general population. The incidence rates of cancers were estimated using number of new cancer cases identified by NHIRD from 1999 to 2007 as numerators and total person-years from individuals with type 2 diabetes and without any diabetes during follow-up period as denominators.

### Statistical analysis

Person-years of two populations were calculated from baseline to the occurrence of specific cancers or closing date (December 31, 2007). SIRs and 95% confidence intervals (CI) were estimated for cancers by using Poisson regression analysis and gender, area registered for NIH program, and age were adjusted. A sensitivity analysis was performed under two conditions. For the first condition, same comparisons were made except in the excluded cancer cases identified in 1999. These cancer cases were excluded because patients are very likely to have cancers at baseline and have not been diagnosed, thereby ruling out the possibility of effect–cause relationship between diabetes and cancer. The second condition included the use of Registry for Catastrophic Illness database to identify cancer cases confirmed by pathological reports to estimate SIRs. PAFs for site-specific cancer incidence caused by diabetes were calculated for each gender by using previously published prevalence estimates of diabetes in Taiwan [2] using the same dataset as the current study with the following formula [26]:

PAF = diabetes prevalence × (RR - 1)/[1 + diabetes prevalence × (RR - 1)]. All statistical analyses were performed using SAS version 9.2 software (SAS Institute, Inc., Cary, NC).

## Results

**Figure 2:**
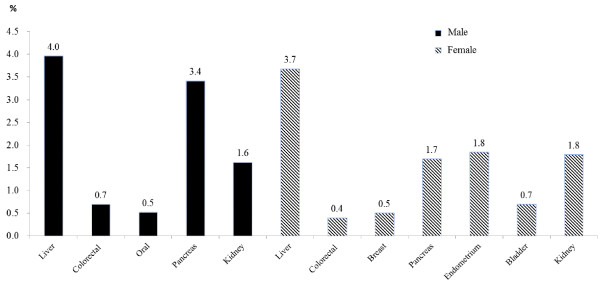
Estimated population attributable fractions (%) for liver, colorectal, oral, pancreas, kidney, breast and endometrium cancer incidence due to type 2 diabetes.

Table 1 shows baseline characteristics of individuals according to type 2 diabetes status stratified by gender in Taiwan. Sex-specific incidence density rates and SIRs for cancer sites from main and sensitivity analyses are shown in Table 2. Cancer sites with increased risks, which were consistent with main and sensitivity analyses, included liver (SIR = 1.61; 95% CI = 1.57 to 1.64 for the main analysis), colorectal (SIR = 1.19; 95% CI = 1.15 to 1.22), oral (SIR = 1.16; 95% CI = 1.12 to 1.21), pancreas (SIR = 1.62; 95% CI = 1.53 to 1.72), and kidney (SIR = 1.32; 95% CI = 1.25 to 1.40) for men. A significant decrease was observed in prostate (SIR = 0.96; 95% CI = 0.93 to 0.99), esophageal (SIR = 0.88; 95% CI = 0.82 to 0.94), and laryngeal (SIR = 0.84; 95% CI = 0.77 to 0.91) cancer incidences for men. For women, cancer sites with increased risks include liver (SIR = 1.55; 95% CI = 1.51 to 1.60 for main analysis), colorectal (SIR = 1.16; 95% CI = 1.13 to 1.20), breast (SIR = 1.14; 95% CI = 1.09 to 1.18), pancreas (SIR = 1.44; 95% CI = 1.34 to 1.55), endometrium (SIR = 1.36; 95% CI = 1.26 to 1.47), bladder (SIR = 1.19; 95% CI = 1.11 to 1.27), and kidney (SIR = 1.38; 95% CI = 1.30 to 1.46). A significant decrease was observed in cervix (SIR = 0.94; 95% CI = 0.91 to 0.99) and connective and other soft tissue (SIR = 0.86; 95% CI = 0.76 to 0.97) cancer incidences. Using Registry for Catastrophic Illness database to identify the cancer cases, SIRs that were not significant, but were significant in main analysis and sensitivity analysis that excluding cancer cases diagnosed within one year of entry included: 1) gallbladder and penile cancers, as well as Hodgkin’s disease, leukemia, and carcinoma in situ in men; and 2) stomach, oral, larynx, and placenta cancers, Hodgkin’s disease, leukemia, and carcinoma in situ in women.Figure 2 shows PAF of site-specific cancer risks that were consistent with main and sensitivity analyses and seemed to have notable associations with diabetes, such as liver, colorectal, oral, pancreas, and kidney for men, and liver, colorectal, breast, pancreas, endometrium, bladder, and kidney for women. These PAFs differed substantially across cancer sites (Figure 2). Overall, PAFs were highest for liver cancer in men (4.0%) and women (3.7%), followed by pancreas (3.4%) and kidney (1.6%) cancers in men, and endometrium (1.8%) and kidney (1.8%) cancers in women.

**Table 1:** Baseline characteristics of individuals according to T2DM status stratified by gender in Taiwan in 1997–1998

	**Gender**			
	**Men**		**Women**	
	**General population (%)**	**Type2 diabetes (%)**	**General population (%)**	**Type2 diabetes (%)**
	**N = 5,185,732**	**N = 233,506**	**N = 4,225,517**	**N = 239,473**
**Age**				
20-24	635,139 (12.25)	543 (0.23)	541,889 (12.82)	532 (0.22)
25-29	853,765 (16.46)	1,543 (0.66)	679,275 (16.08)	1,605 (0.67)
30-34	836,516 (16.13)	4,150 (1.78)	676,492 (16.01)	3,039 (1.27)
35-39	752,396 (14.51)	9,642 (4.13)	622,116 (14.72)	5,366 (2.24)
40-44	615,146 (11.86)	17,613 (7.54)	500,234 (11.84)	10,357 (4.32)
45-49	431,072 (8.31)	25,831 (11.06)	342,065 (8.10)	18,001 (7.52)
50-54	240,445 (4.64)	24,387 (10.44)	195,267 (4.62)	23,547 (9.83)
55-59	221,643 (4.27)	29,662 (12.70)	192,694 (4.56)	34,299 (14.32)
60-64	176,070 (3.4)	31,195 (13.36)	148,673 (3.52)	39,859 (16.64)
65-69	171,633 (3.31)	36,445 (15.61)	114,456 (2.71)	40,252 (16.81)
70-74	124,652 (2.40)	28,984 (12.41)	84,367 (2.00)	31,256 (13.05)
75-79	72,715 (1.40)	15,164 (6.49)	60,737 (1.44)	18,873 (7.88)
80-84	37,193 (0.72)	6,148 (2.63)	39,697 (0.94)	8,721 (3.64)
85-89	13,621 (0.26)	1,829 (0.78)	20,066 (0.47)	3,087 (1.29)
≥90	3,726 (0.07)	370 (0.16)	7,489 (0.18)	679 (0.28)
**Insurance premiums**				
<19200	1,615,639 (31.16)	75,398 (32.29)	1,339,863 (31.71)	61,621 (25.73)
≥19200	3,570,093 (68.84)	158,108 (67.71)	2,885,654 (68.29)	177,852 (74.27)
**Urbanization degree of area registered for National Health Insurance program**				
High density urban area	1,551,499 (30.28)	63,914 (27.64)	1,398,129 (33.52)	61,109 (25.82)
Medium density urban area	1,433,981 (27.99)	67,054 (29.00)	1,183,288 (28.37)	64,685 (27.33)
Newly developed area	985,111 (19.23)	39,778 (17.20)	719,525 (17.25)	38,390 (16.22)
General area	697,202 (13.61)	35,137 (15.20)	534,267 (12.81)	40,501 (17.11)
Aging society area	90,159 (1.76)	6,204 (2.68)	69,560 (1.67)	7,877 (3.33)
Rural area	187,136 (3.65)	10,345 (4.47)	138,781 (3.33)	13,034 (5.51)
Non-developed area	178,634 (3.49)	8,803 (3.81)	127,110 (3.05)	11,096 (4.69)

**Table 2:** Gender-specific incidence density rates and SIRs for cancer sites

	**General population**^**(a)**^		**Population with type2 diabetes**		**Main analysis using outpatient and inpatient databases**		**After excluding cancer cases diagnosed within 1 year of entry**^**(b)**^		**Using Registry for Catastrophic Illness Database**^**(c)**^	
**Type of cancer**	**O**	**Incidence density**^**#**^	**O**	**Incidence density**^**#**^	**SIR**	**95% CI**	**SIR**	**95% CI**	**SIR**	**95% CI**
***Men***										
**Lung**	53072	1.14	7493	3.64	0.99	(0.97-1.02)	**0.96**	(0.94-0.99)**	**0.74**	(0.72-0.77)***
**Liver**	64321	1.39	9375	4.57	**1.61**	(1.57-1.64)***	**1.59**	(1.55-1.62)***	**1.70**	(1.65-1.76)***
**Colorectal**	46715	1.01	6048	2.92	**1.19**	(1.15-1.22)***	**1.16**	(1.13-1.20)***	**1.12**	(1.07-1.17)***
**Stomach**	18918	0.41	2528	1.21	**0.96**	(0.92-1.00)*	0.96	(0.92-1.00)	**0.89**	(0.85-0.95)***
**Oral**	30942	0.67	2411	1.15	**1.16**	(1.12-1.21)***	**1.15**	(1.10-1.21)***	**1.09**	(1.03-1.15)**
**Prostate**	27767	0.60	4259	2.05	**0.96**	(0.93-0.99)*	**0.94**	(0.91-0.97)***	**0.89**	(0.85-0.94)***
**Esophagus**	11667	0.25	1073	0.51	**0.88**	(0.82-0.94)***	**0.88**	(0.82-0.94)***	**0.69**	(0.63-0.75)***
**Pancreas**	7023	0.15	1337	0.64	**1.62**	(1.53-1.72)***	**1.56**	(1.46-1.67)***	**1.60**	(1.45-1.77)***
**Cervix**	-	-	-	-	-	-	-	-	-	-
**Nasopharyngeal**	20022	0.43	1251	0.60	**1.09**	(1.03-1.16)***	1.04	(0.98-1.11)	**0.85**	(0.76-0.95)**
**Small intestine**	2129	0.05	287	0.14	**1.15**	(1.02-1.31)*	1.11	(0.97-1.28)	0.82	(0.64-1.05)
**Gallbladder**	4522	0.10	693	0.33	**1.18**	(1.08-1.28)***	**1.15**	(1.05-1.25)**	1.10	(0.97-1.25)
**Retroperitoneum and peritoneum**	1274	0.03	160	0.08	**1.19**	(1.00-1.40)*	1.14	(0.95-1.37)	1.06	(0.74-1.51)
**Larynx**	6821	0.15	596	0.28	**0.84**	(0.77-0.91)***	**0.83**	(0.79-0.91)***	**0.75**	(0.65-0.85)***
**Respiratory and intrathoratic organs**	7399	0.16	745	0.36	0.93	(0.86-1.00)	**0.90**	(0.83-0.98)*	**0.81**	(0.65-1.00)*
**Bone**	3778	0.08	350	0.17	0.93	(0.83-1.04)	0.90	(0.79-1.01)	0.90	(0.64-1.26)
**Connective and other soft tissue**	4536	0.10	428	0.20	1.07	(0.96-1.18)	1.06	(0.95-1.18)	0.81	(0.64-1.03)
**Skin**	1605	0.03	199	0.09	1.10	(0.95-1.28)	1.08	(0.92-1.27)	1.10	(0.85-1.43)
**Testis**	2017	0.04	127	0.06	1.16	(0.96-1.40)	1.18	(0.97-1.44)	0.71	(0.34-1.47)
**Penis**	1132	0.02	148	0.07	**1.88**	(1.57-2.25)***	**1.41**	(1.16-1.71)***	1.32	(0.96-1.82)
**Bladder**	12758	0.27	1802	0.86	**1.07**	(1.02-1.13)**	1.04	(0.99-1.10)	0.99	(0.92-1.06)
**Kidney**	9913	0.21	1504	0.72	**1.32**	(1.25-1.40)***	**1.30**	(1.22-1.38)***	**1.28**	(1.17-1.40)***
**Brain**	7596	0.16	732	0.35	1.03	(0.96-1.12)	0.99	(0.91-1.08)	**0.77**	(0.65-0.92)**
**Hodgkin’s disease**	760	0.02	106	0.05	**1.69**	(1.37-2.10)***	**1.42**	(1.11-1.81)**	1.05	(0.63-1.73)
**Leukemia**	4917	0.11	928	0.44	**1.86**	(1.73-2.00)***	**1.66**	(1.54-1.80)***	0.96	(0.84-1.09)
**Carcinoma in situ**	11323	0.24	1251	0.60	**1.18**	(1.11-1.25)***	**1.15**	(1.07-1.23)***	0.81	(0.18-3.58)
***Women***										
**Lung**	24872	0.66	4498	2.11	0.98	(0.95-1.02)	0.97	(0.94-1.00)	**0.74**	(0.70-0.78)***
**Liver**	28577	0.75	6061	2.85	**1.55**	(1.51-1.60)***	**1.54**	(1.49-1.58)***	**1.67**	(1.60-1.75)***
**Colorectal**	33469	0.88	5502	2.59	**1.16**	(1.13-1.20)***	**1.15**	(1.11-1.18)***	**1.07**	(1.01-1.12)***
**Breast**	42441	1.12	2934	1.37	**1.14**	(1.09-1.18)***	**1.13**	(1.09-1.18)***	**1.09**	(1.04-1.14)***
**Stomach**	9632	0.25	1830	0.85	**1.11**	(1.05-1.17)***	**1.12**	(1.06-1.18)***	1.05	(0.98-1.13)
**Oral**	4970	0.13	733	0.34	**1.30**	(1.20-1.42)***	**1.27**	(1.16-1.39)***	1.08	(0.94-1.25)
**Esophagus**	7500	0.20	648	0.30	1.08	(0.99-1.18)	1.06	(0.97-1.16)	**0.70**	(0.54-0.90)**
**Pancreas**	4700	0.12	1071	0.50	**1.44**	(1.34-1.55)***	**1.42**	(1.32-1.53)***	**1.31**	(1.17-1.46)***
**Cervix**	27472	0.73	2579	1.21	**0.94**	(0.90-0.99)**	**0.93**	(0.89-0.98)**	**0.84**	(0.79-0.90)***
**Nasopharyngeal**	8789	0.23	690	0.32	**1.14**	(1.05-1.24)**	1.09	(0.99-1.19)	0.98	(0.82-1.18)
**Small intestine**	1248	0.03	222	0.10	1.11	(0.96-1.29)	1.04	(0.88-1.22)	1.04	(0.81-1.34)
**Gallbladder**	3278	0.09	697	0.32	**1.10**	(1.01-1.20)*	1.08	(0.99-1.19)	1.08	(0.96-1.22)
**Retroperitoneum and peritoneum**	1358	0.04	201	0.09	1.16	(0.99-1.35)	1.13	(0.95-1.33)	1.16	(0.89-1.52)
**Larynx**	1272	0.03	137	0.06	**1.30**	(1.07-1.57)**	**1.35**	(1.10-1.66)**	1.41	(0.87-2.27)
**Respiratory and intrathoratic organs**	3889	0.10	495	0.23	0.94	(0.85-1.04)	0.92	(0.83-1.02)	**0.66**	(0.50-0.88)**
**Bone**	2555	0.07	333	0.15	1.03	(0.91-1.16)	1.00	(0.88-1.14)	0.78	(0.52-1.18)
**Connective and other soft tissue**	3284	0.09	309	0.14	**0.86**	(0.76-0.97)*	**0.84**	(0.73-0.95)**	**0.76**	(0.58-0.99)*
**Skin**	1265	0.03	189	0.09	1.10	(0.94-1.29)	0.99	(0.83-1.18)	0.93	(0.71-1.22)
**Placenta**	450	0.01	385	0.18	**19.90**	(16.40-24.16)***	**8.74**	(6.65-11.48)***	1.59	(0.44-5.74)
**Endometrium**	8094	0.21	835	0.39	**1.36**	(1.26-1.47)***	**1.28**	(1.18-1.39)***	**1.32**	(1.17-1.49)***
**Ovary**	13406	0.35	873	0.41	1.02	(0.95-1.10)	1.02	(0.94-1.10)	**0.85**	(0.75-0.96)**
**Bladder**	6043	0.16	1151	0.54	**1.19**	(1.11-1.27)***	**1.17**	(1.09-1.25)***	**1.13**	(1.03-1.25)*
**Kidney**	6943	0.18	1471	0.68	**1.38**	(1.30-1.46)***	**1.36**	(1.28-1.45)***	**1.32**	(1.21-1.45)***
**Brain**	6370	0.17	791	0.37	**1.15**	(1.06-1.25)***	**1.10**	(1.01-1.20)*	**0.80**	(0.66-0.97)*
**Hodgkin’s disease**	543	0.01	91	0.04	**1.84**	(1.44-2.35)***	**1.82**	(1.38-2.39)***	1.35	(0.64-2.87)
**Leukemia**	3353	0.09	830	0.39	**2.11**	(1.94-2.29)***	**1.84**	(1.68-2.03)***	0.96	(0.83-1.12)
**Carcinoma in situ**	49189	1.30	3142	1.47	**0.93**	(0.90-0.97)***	**0.92**	(0.88-0.96)***	1.05	(0.36-3.06)

(^a^) The SIR for general population is 1.00.

(^b^) Observed number of cancer cases for population with type 2 diabetes after exclusion of cancers diagnosed within 1 year of cohort entry.

(^c^) Observed number of cancer cases for population with type 2 diabetes using Registry of Catastrophic Illness Database

SIRs were adjusted for covariates (insurance premium, urbanization degree of area registered for National Health Insurance program, and age).

Bold type indicates that the 95% CI does not include 1.00. *Abbreviations*: *CI* confidence interval, *O* observed number of cancer cases, *SIR* standardized incidence ratio. #: per 1000 person-years; *: p < 0.05; **: p < 0.01; ***: p < 0.001.

## Discussion

To the best of our knowledge, this report is the largest study to examine SIRs and PAFs of diabetes on site-specific cancer incidence for Taiwanese population. This nationwide population-based cohort study included 474,686 patients with type 2 diabetes whose ages were ≥ 20 years at baseline. General population consists of approximately 10 million individuals who enrolled in NHI program with the same age limits but with no diabetes. All individuals in this study have been followed up for 10 to 11 years. In this retrospective nationwide population study, a diagnosis of DM was associated with 61%, 19%, 16%, 62%, and 32% increases in risks of liver, colorectal, oral, pancreatic, and kidney cancer incidences in Taiwanese men, respectively. A similar result was also observed in women, in which 55%, 16%, 14%, 44%, 36%, 19%, and 38% increases in liver, colorectal, breast, pancreas, endometrium, bladder, and kidney cancers were observed, respectively. This study showed similarity in magnitude of risks between men and women. Our study provided estimates for site-specific cancer risks for Taiwanese with type 2 diabetes by adjusting for population structure. In particular, association between diabetes and oral cancer has never been reported. Furthermore, proportions of total risks for site-specific cancers in Taiwanese population that can be attributed to type 2 diabetes were estimated using the entire populations with and without type 2 diabetes.

Studies on the relationship between diabetes and cancer using SIRs have indicated that diabetes has an increased risk of liver [6], colon [5,6,18], pancreas [6], esophagus [6], stomach [6], and lung [6], cancers, whereas risk of prostate cancer is lower [6]. Diabetes is also associated with higher risk of breast cancer according to several studies [6]. By contrast, other studies have shown that diabetes is associated with lower risk for breast cancer [27]. The findings regarding increased risks of liver, colorectal, pancreatic, and kidney cancers are consistent with those in previous studies [28-34]. We also observed higher risks of breast, bladder, and endometrium cancers in women, which is consistent with findings from previous studies [19]. A significant inverse association between diabetes and prostate cancer has been observed in men, which is also consistent with previous epidemiological studies [19,35-38], but inconsistent with those that show no associations [30-34,39]. At the other sites, we found a negative association for esophageal and laryngeal cancers in males, as well as for cervical and connective and other soft tissue cancers in females. However, previous epidemiological studies [30-34,40,41] have found no evidence for an association with these cancers, although several studies have shown negative associations [19].

Our study, along with previous studies, indicated that diabetes is a risk factor for cancers. Many possible biological mechanisms are involved in the association between DM and overall or a specific cancer. Diabetes may influence cancer by hyperinsulinemia, hyperglycemia, or inflammation as a result of metabolic and hormonal aberrations [17]. Diabetic individuals normally have hyperinsulinemia and are associated with reduced insulin sensitivity and compensatory hyperinsulinemia as well as increased insulin-like growth factor (IGF)-1 levels, which may stimulate cell proliferation in liver, pancreas, colon, ovary, breast, and other areas. Insulin and IGFs may promote tumor cell growth, which increases risk of cancers. Among cancers that we have studied, liver and pancreatic cancers were the two types that exhibited the highest SIRs associated with type 2 diabetes. Insulin is produced by pancreatic β cells through hepatic portal vein to liver, which, along with pancreas, is exposed to high insulin concentrations [17]. Considering inflammatory function of insulin, previous studies have shown a strong association between obesity and diabetes [15]. Obesity may increase risk of cancers because obese individuals have higher levels of leptin and lower levels of serum adiponectin [16], which is associated with chronic inflammation [42]. Association between DM and cancer can also be associated with the changes in sex hormone levels that occur in several types of cancer, such as prostate cancer. Testosterone affects the growth of prostate gland [43]; in particular, a high testosterone level is associated with prostate cancer [44]. Previous studies have also indicated that diabetic men have lower testosterone levels [45], which suggest a decreased risk in prostate cancer. Thus, decreased risk observed in this study is biologically plausible.

Our sensitivity analysis showed that estimated SIRs of many major cancers were similar to those from the analysis, in which cancer cases identified in 1999 were excluded as well as cancer cases obtained from Registry for Catastrophic Illness database, except for stomach cancer. These consistent findings showed that the results of our study were robust. For several cancers with lower incidence rates, such as nasopharyngeal, small intestine, and brain cancers, SIR estimates based on Registry for Catastrophic Illness database are not consistent with those in the other two methods. The possible explanation for this inconsistency is that our sample size is not large enough for such low incidence rates that SIR estimates are not reliable enough. To be conservative, we only discussed cancer types with SIRs that are consistent with main and sensitivity analyses.

Our study showed men with a diagnosis of type 2 DM were associated with increases in risks of liver, colorectal, oral, pancreatic, and kidney cancer incidences and women with a diagnosis of type 2 diabetes with increases in liver, colorectal, breast, pancreas, endometrium, bladder, and kidney cancers. These findings have important clinical implication: it is necessary to develop strategies of cancer-specific screening and prevention care in patients with type 2 diabetes for men and women. For future studies, what factors are associated with increased or decreased risks of site-specific cancer in patients with type 2 diabetes needs further investigation. In term of public health implication, we estimate that number of incident cases of liver, colorectal, pancreatic, and kidney cancers for men that can be attributable to type 2 diabetes by 272, 50, 194, 28, and 8, respectively; number of incident cases of liver, colorectal, breast, pancreatic, bladder, and kidney cancers for women by 105, 21, 50, 11, 4, and 5, respectively, based on number of incident cases from Taiwan National Registry for Cancer in 2010 and SIRs and PAFs of type 2 diabetes indicated in our study. These findings provide information for health policy makers on evaluation of the cost-effectiveness of cancer screening and prevention program.

### Strengths and limitations

This study has several merits. First, this study is considered a large study that involved estimation of SIRs for cancer patients with type 2 diabetes. Thus, this study has sufficient capability to detect the effect of type 2 diabetes and to adjust according to several risk factors, such as age, gender, insurance premium, and urbanization degree of area registered for NIH program through standardization. Although Asia Pacific Cohort Studies Collaboration (APCSC) has examined associations between diabetes and cancer mortality with a large sample size (Lam et al., [28]), our study has two advantages. One is that participants of APCSC are from thirty-six cohort Asian and Australasian studies with various ethnic origins, which may modify associations between diabetes and cancer incidences. The other is that APCSC has focused on cancer mortality, and cancer incidence has not been considered. Second, NHIRD included all diagnosed records. Thus, we can accurately determine cancer incidence and minimize the number of subjects in the cohort who were lost during follow-up period. Third, data with one-year left-censored for exploring the possibility of reverse causality had a negligible effect on original estimates. In addition, most of estimated SIRs are similar to those obtained from analysis, in which cancer incidences obtained from Registry for Catastrophic Illness database were used. The consistent findings from our sensitivity analysis indicated that our results are robust.

Several limitations of the study were also observed. First, we cannot obtain data of behavioral factors, such as smoking, alcohol consumption, obesity, body mass index, and physical activity. In addition, we cannot determine familial risks for diabetes to explain effects of genetic and environmental factors. Thus, independent effect of type 2 diabetes on cancer cannot be established. However, our study allows for rate comparison by adjusting for population structure of age, gender, insurance premium, and area registered for NIH program, which can be performed as the first step of this line of research. Second, diabetic patients may have taken medicine that affected cancer risks. Previous studies have also indicated that glucose-lowering medicines, such as metformin, may reduce risks of cancers in diabetic patients. On the contrary, sulfonylurea drugs or insulin are associated with increased cancer risks [46]. Thus the strength of association between type 2 diabetes and cancer estimated for different populations depend on prevalence of anti-diabetes medication in population with diabetes. Although we did not have information regarding glucose-lowering medications, it won’t confound our estimation for association between type 2 diabetes and cancer in our population.

## Conclusion

Our data suggested that unusual risks of cancer are associated with type 2 diabetes. Significant increased risks were observed in liver, colorectal, oral, pancreatic, and kidney cancers in men, and in liver, colorectal, breast, pancreatic, endometrium, bladder, and kidney cancers in women. Reduced risks were observed in prostate, esophageal, and laryngeal cancers in men. Reduced risks were also found in cervical and connective and other soft tissue cancers in women.

## Competing interests

The authors declare that they have no competing interests.

## Authors’ contributions

TCL, JHC and CCL contributed equally to the design of the study and the direction of its implementation, including supervision of the field activities, quality assurance and control. CIL, CSL, and WYL supervised the field activities. CSL, CCL, TFH and CIL helped conduct the literature review and prepare the Methods and the Discussion sections of the text. TCL and JHC designed the study’s analytic strategy and conducted the data analysis. All authors read and approved the final manuscript.

## Pre-publication history

The pre-publication history for this paper can be accessed here:

http://www.biomedcentral.com/1471-2407/14/381/prepub
